# Maternal/zygotic knockout and droplet digital polymerase chain reaction analysis question the role of activin A in preimplantation mouse embryo development^†^

**DOI:** 10.1093/biolre/ioaf189

**Published:** 2025-07-22

**Authors:** Eliza Winek, Katarzyna Szczepańska, Lidia Wolińska-Nizioł, Katarzyna Zgódka, Michaela Vaskovicova, David Drutovic, Aneta Suwińska

**Affiliations:** Department of Embryology, Institute of Developmental Biology and Biomedical Sciences, Faculty of Biology, University of Warsaw, Warsaw, Poland; Department of Embryology, Institute of Developmental Biology and Biomedical Sciences, Faculty of Biology, University of Warsaw, Warsaw, Poland; Department of Embryology, Institute of Developmental Biology and Biomedical Sciences, Faculty of Biology, University of Warsaw, Warsaw, Poland; Department of Embryology, Institute of Developmental Biology and Biomedical Sciences, Faculty of Biology, University of Warsaw, Warsaw, Poland; Laboratory of DNA Integrity, Institute of Animal Physiology and Genetics of the Czech Academy of Sciences, Libechov, Czech Republic; Laboratory of DNA Integrity, Institute of Animal Physiology and Genetics of the Czech Academy of Sciences, Libechov, Czech Republic; Department of Embryology, Institute of Developmental Biology and Biomedical Sciences, Faculty of Biology, University of Warsaw, Warsaw, Poland

**Keywords:** activin A, Inhba, preimplantation embryo, maternal knockout, ddPCR

## Abstract

For many years, activin A, encoded by *Inhba,* has been thought to be present in both mouse and human oocytes and preimplantation embryos. However, its deficiency does not impede the proper embryonic development of the embryo until birth. It has been suggested that the lack of a phenotype in zygotic knockout embryos may be masked by the presence of maternal protein deposited in the oocyte during oogenesis or provided from the reproductive tract. Therefore, to explore whether maternally supplied activin A is required for embryo development, we carried out a conditional *Inhba* knockout in oocytes using *Zp3*-Cre/LoxP strategy. By examining *Inhba* maternal and maternal/zygotic knockout embryos, individually recorded using time-lapse imaging, immunostained, and genotyped, we revealed that the maternal pool of activin A affects the dynamics of mouse preimplantation development. These alterations are accompanied by impaired mitochondrial activity in oocytes. Surprisingly, using the droplet digital polymerase chain reaction approach, we provided evidence that the *Inhba* mRNA of zygotic origin is undetectable in mouse embryos.

## Teaser

This study challenges the existing knowledge regarding the expression pattern and role of activin A in the preimplantation development of the mouse embryo.

## Graphical Abstract

**Graphical Abstract:**
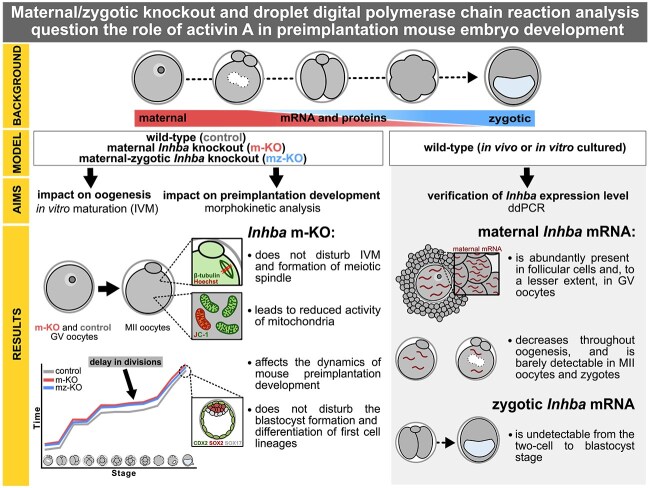

## Introduction

activin A, a member of the transforming growth factor β family, has been detected in oocytes and at various preimplantation stages in both mouse and human embryos [1–5]. However, there are discrepancies in the literature regarding the expression pattern of activin A during mouse embryogenesis. Using immunofluorescence, Albano et al. reported that activin A protein levels were highest in the zygote, decreased at the two- and four-cell stages, and then increased again at the eight-cell stage, morula and blastocyst [1]. Depending on the detection method, *Inhba* mRNA has been reported in mouse oocytes and embryos from the 2-cell to the blastocyst stage [5], in oocytes and the morula stage [1], in the oocytes and embryos up to the 8-cell stage [4] or exclusively at the blastocyst stage [3]. More recently, transcriptomic and proteomic data have yielded similarly inconsistent results, showing undetectable to low levels of *Inhba* mRNA and protein in mouse oocytes and preimplantation embryos [6–9]. These discrepancies highlight a critical gap in our understanding of both the expression pattern and the functional role of activin A during early development. Because *Inhba* belongs to a low-abundance targets, using more sensitive and targeted detection methods may be crucial to resolve these inconsistencies.

Notably, embryonic phenotypes resulting from loss-of-function mutations in components of activin A pathway are not observed before implantation [10–12]. Knockouts of zygotic activin A develop normally until the late blastocyst stage [13, 14] and display developmental abnormalities only after birth [12]. Moreover, their successful preimplantation development is not dependent on the exogenous activin A, which is produced by the reproductive system organs [1, 3, 4] as activin A-deficient embryos cultured in vitro also develop without disruption until the blastocyst stage [14]. However, it has been suggested that the lack of a notable phenotype during the preimplantation period of development may result from the potential rescue of zygotic deficiencies by proteins of maternal origin.

It is known that before zygotic genome activation (ZGA), embryonic development relies on maternally inherited RNAs and proteins accumulated during oogenesis [15–17]. In mice, ZGA begins during the late 1-cell stage (minor ZGA), followed by major gene activation at the 2-cell stage. After that stage, the gradual degradation of maternal gene products accompanies the onset of transcription from the zygote’s genome. However, maternal mRNA may persist until the blastocyst stage, potentially rescuing or delaying the manifestation of mutant phenotypes in zygotic knockout embryos. Therefore, we genetically ablated maternal activin A, as well as simultaneously maternal and zygotic activin A in the embryos, and followed their development to the blastocyst stage in vitro*.* This approach not only eliminated endogenous activin A, but also excluded any influence of exogenous activin A provided by the maternal reproductive system. We found that while embryos deprived of maternal activin A were phenotypically indistinguishable from control blastocysts, they displayed retarded course of preimplantation development correlated with mitochondrial dysfunction in oocytes. Furthermore, given the absence of a preimplantation phenotype in zygotic knockout embryos [14] and the conflicting previous reports regarding the presence of this growth factor before implantation, we decided to verify the mRNA expression levels of *Inhba* in oocytes and during subsequent stages of mouse preimplantation development. We used droplet digital polymerase chain reaction (ddPCR) technology, which enables highly sensitive quantification of low transcript levels. Nevertheless, we did not detect *Inhba* mRNA of zygotic origin at any preimplantation stage, thereby resolving inconsistencies in the literature. Our findings challenge the current knowledge about *Inhba* gene expression and the role of activin A protein in preimplantation embryonic development in mice.

## Results

### Mouse model

**Figure 1:**
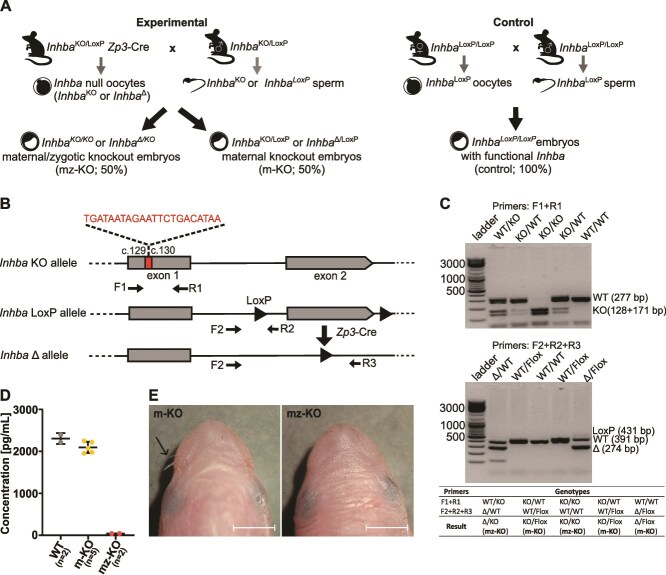
Mouse model with maternal/zygotic knockout (mz-KO) of activin A gene. (A) Breeding schemes used to obtain the experimental groups: *Inhba* maternal/zygotic knockout (mz-KO), maternal knockout embryos (m-KO), and control - *Inhba*^LoxP/LoxP^ embryos. (B) Scheme of the mz-KO mutation, created by crossing three mouse lines: one with activin A gene knockout (*Inhba*-KO allele), a second with the floxed but functional second exon of the *Inhba* gene *(Inhba-*LoxP*),* and a *Zp3*-Cre mouse line. *Inhba*-KO mutation involving cassette insertion between the 29th and 30th nucleotides of the first exon of the *Inhba* gene. The cassette contains the STOP codon and EcoRI enzyme digestion sequences, leading to a frameshift mutation and premature translation termination. On the second allele, the floxed second exon of the *Inhba* gene was excised by the *Zp3*-dependent Cre recombinase in growing oocytes, resulting in a null allele, referred to as *Inhba* Δ. Forward and reverse primers used for genotyping are marked in the scheme. (C) Representative genotyping results showing the knockout, floxed, and null alleles, allowing to distinguish between maternal-knockout (m-KO) and maternal/zygotic knockout (mz-KO) embryos. (D) Levels of activin A protein in the culture medium of MEFs, measured by the ELISA method. Data are presented as medians with interquartile ranges. (E) The phenotype of *Inhba* mutant mice. The arrow in the photo of m-KO mouse shows the whiskers, which are absent in mz-KO mice. Scale bars: 2 mm.

To investigate the effect of maternal-origin activin A on preimplantation mouse embryo development, we used the CRISPR/Cas9 method to obtain embryos with a knockout of the maternal activin A (*Inhba*^KO/LoxP^ and *Inhba*^Δ/LoxP^; henceforth referred to as *Inhba* m-KO), and maternal and zygotic protein (*Inhba*^KO/KO^ and *Inhba*^Δ/KO^; henceforth referred to as *Inhba* mz-KO; Figure 1A). The *Inhba* mz-KO mutation was created by crossing three mouse lines: *Inhba*-KO with activin A gene knockout (*Inhba*-KO allele; described previously in [14]), the *Inhba*-LoxP line with a floxed but functional second exon of *Inhba* gene (*Inhba-*LoxP allele)*,* and the *Zona pellucida 3 (Zp3)*-Cre mouse line, where Cre expression is controlled by regulatory sequences from the mouse *Zp3* gene [18]. *Zp3*-Cre-mediated recombination of exon 2 of the *Inhba* gene results in a null allele (*Inhba* Δ), allowing conditional depletion of activin A, specifically in growing oocytes, prior to the completion of the first meiotic division (Figure 1B).

We confirmed the presence of knockout, floxed, and recombined null alleles, using PCR assays to distinguish *Inhba* m-KO embryos from *Inhba* mz-KO embryos (Figure 1C). Furthermore, the absence of activin A protein in *Inhba* mz-KO embryos was demonstrated in the ELISA assay of the culture medium from mouse embryonic fibroblasts (MEFs) derived from *Inhba* mz-KO, *Inhba* m-KO and wild-type fetuses (Figure 1D).

To demonstrate that Cre-mediated recombination impairs the function of the *Inhba* gene, we intercrossed an *Inhba*^KO/LoxP^  *Zp3*-Cre female with an *Inhba*^KO/LoxP^ male and dissected the resulting embryos at 19 days post coitum. We confirmed that *Inhba* m-KO fetuses developed normally, whereas *Inhba* mz-KO fetuses displayed a characteristic phenotype: the absence of whiskers and lower incisors, consistent with findings from previous studies using independently derived zygotic knockout lines [12, 14]; (Figure 1E). Hence, the results obtained confirm the validity of using our mouse line to study the effects of maternal activin A on mouse embryogenesis.

### The lack of maternal activin A does not impair the oocyte in vitro maturation process

To determine whether maternal activin A depletion affects oocyte in vitro maturation (IVM), we isolated fully grown germinal vesicle (GV) stage oocytes from unstimulated with exogenous gonadotropins *Inhba*^KO/LoxP^  *Zp3*-Cre and control *Inhba*^LoxP/LoxP^ females. The ability of the oocytes to reach the metaphase II (MII) stage was evaluated 16 h post-isolation, based on the extrusion of the first polar body and GV breakdown.

**Figure 2:**
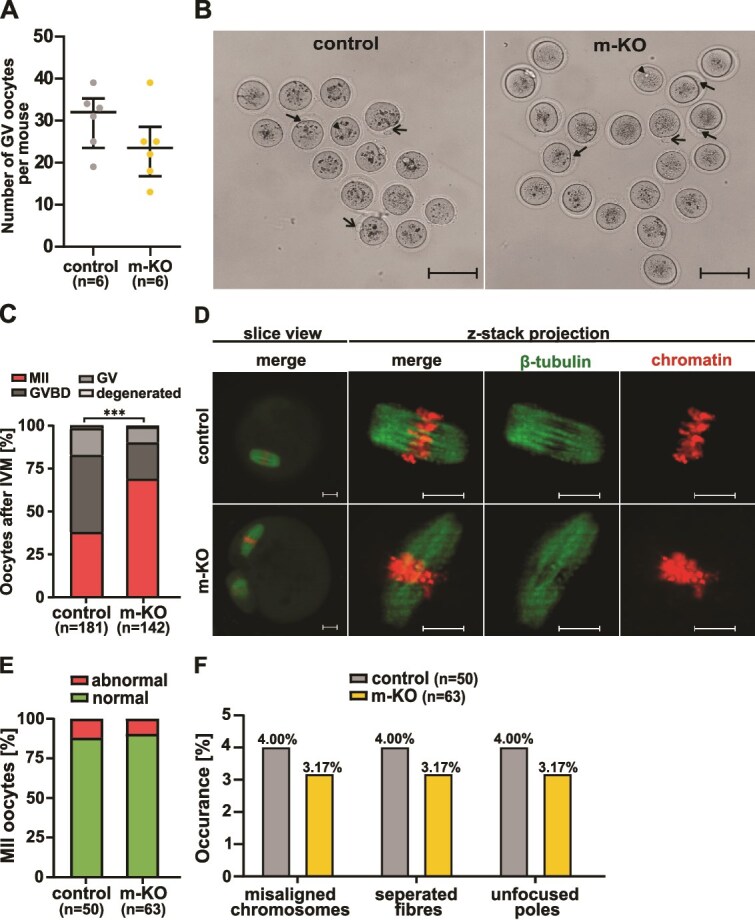
The lack of maternal activin A in oocytes does not disturb IVM. (A) The yield of control (from *Inhba*^LoxP/LoxP^ female) and m-KO (from *Inhba*^KO/LoxP^  *Zp3*-Cre female) oocytes, represented as the number of GV oocytes isolated from one female (*n* = 6 females in each group). Data are presented as median and the values of the first and third quartiles. Statistical analysis using the Mann–Whitney U-test confirmed no significant changes (*P* > 0.05) between groups. (B) Bright-field images of control and m-KO oocytes after 16 h of IVM. Open arrows indicate the ejected first polar body, whereas closed arrows point to its fragmented remnants, confirming the successful maturation of oocytes to the metaphase II stage (MII stage). Arrowheads show immature GV oocytes with visible GVs. Scale bar: 100 μm. (C) Proportions of control and m-KO oocytes after 16 h of IVM, categorized into mature MII stage oocytes (MII), immature oocytes: after GV breakdown (GVBD) and in the GV stage, and degenerated oocytes. The total number of oocytes from six independent experiments is noted in parentheses on the graph. Statistical analysis using Fisher Exact test revealed a significant difference in maturation efficiency (^***^P < 0.001). (D) Representative images of MII meiotic spindles after IVM of control and m-KO oocytes. The meiotic spindle structures and chromosome alignment were assessed based on z-stack projections after β-tubulin and chromatin staining. Scale bars: 10 μm. (E) Percentages of MII stage oocytes with abnormal versus normal spindle morphology. Chromosomes and spindle malformations were identified in five independent experiments, and the total number of analyzed control and m-KO oocytes is indicated in parentheses. Statistical comparison using the Fisher Exact test revealed no significant differences (*P* > 0.05) between control and m-KO groups. (F) The percentages of MII-stage oocytes with the misaligned chromosomes dispersed outside the metaphase plate, fibers separated from the spindle, and unfocused spindle poles. Fisher Exact test conducted separately for each parameter confirmed no significant differences (*P* > 0.05) between control and m-KO groups.

We noted that the knockout of maternal activin A did not affect the yield of GV oocytes, as the median number of isolated GV oocytes per female was similar in the control and *Inhba* m-KO groups (*P* > 0.05; Mann–Whitney U-test; *n* = 6 females per group; Figure 2A). Furthermore, the *Inhba* m-KO oocytes did not exhibit any defects in GV breakdown (GVBD) or first polar body extrusion (Figure 2B). On the contrary, after IVM, we observed a higher proportion of mature oocytes in *Inhba* m-KO than in the control group. Approximately 69.01% (98/142) of *Inhba* m-KO oocytes reached the MII stage, whereas in the control group this percentage amounted to 38.12% (69/181; *P* < 0.001; Fisher Exact test; Figure 2C). We then used chromatin and β-tubulin labeling to assess the potential effects of maternal activin A depletion on meiotic spindle formation and associated chromosome configuration. Spindle organization was classified as morphologically normal if all microtubules were concentrated at the opposite spindle poles and chromosomes were aligned on the metaphase plate. Abnormal spindle structures included protrusion of microtubules from the spindle, disorganized spindle poles, and dispersed chromosomes outside the metaphase plate. Based on these criteria, we observed that the majority of MII-stage oocytes from both the control group (44/50) and *Inhba* m-KO group (57/63) exhibited properly formed meiotic spindles (Figure 2D). Notably, the percentage of oocytes with abnormal meiotic spindles was similar in the control (12%; 6/50) and *Inhba* m-KO group (9.52%; 6/63; *P* > 0.05; Fisher Exact test; Figure 2E). Similarly, the percentage of oocytes with abnormalities in chromosomes arrangement, microtubule organization, and spindle poles structure was comparable in both analyzed groups (control: 4%; 2/50 for each parameter; *Inhba* m-KO: 3.17%; 2/63 for each parameter; *P* > 0.05; Fisher Exact test for all analyses; Figure 2F).

These findings suggest that the absence of maternal activin A does not affect the formation of the second meiotic spindle and the efficiency of the oocyte IVM process.

### Maternal and maternal/zygotic *Inhba* knockout embryos display retarded preimplantation development despite being phenotypically indistinguishable

**Figure 3:**
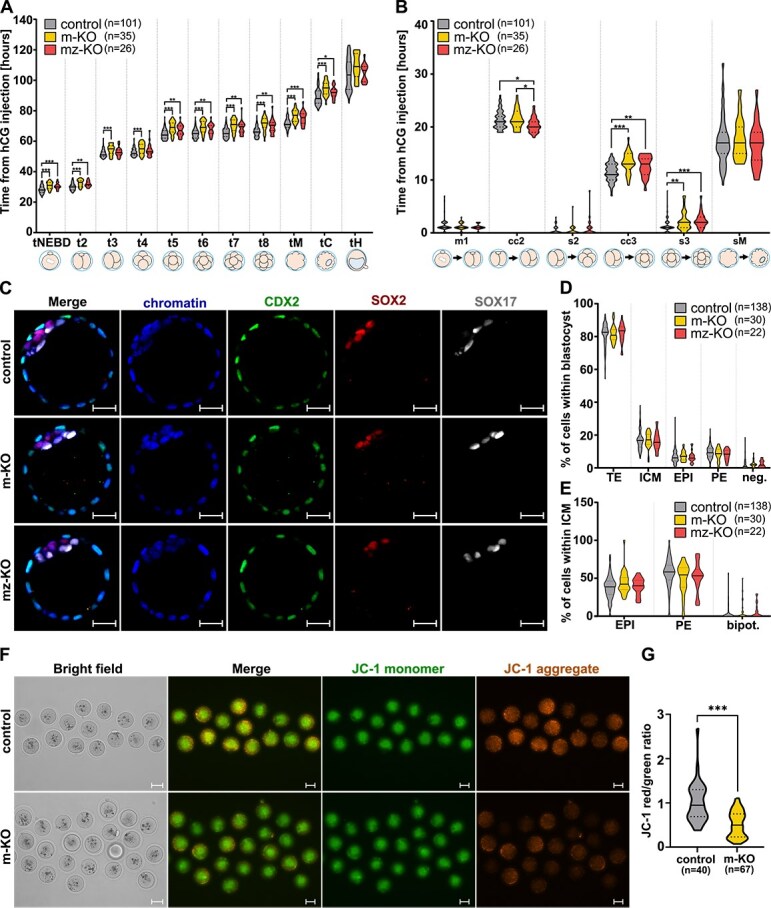
Embryos deprived of maternal activin A exhibit delayed development to the blastocyst stage and compromised mitochondrial activity. Comparison of main morphokinetic parameters between maternalknockout (m-KO), maternal/zygotic knockout (mz-KO), and control – *Inhba*^LoxP/LoxP^ embryos. (A) These parameters were measured as the time (in hours) from hCG injection to specific developmental milestones: tNEBD—breakdown of nuclear envelopes of pronuclei; t2–t8—completion of division to the respective number of blastomeres; tM—compaction; tC and tH—initiation of cavitation and hatching process, respectively. (B) The value of individual parameters was calculated as the number of hours between: m1 – the disappearance of pronuclei and 2-cell stage, i.e., duration of the first embryonic M-phase; cc2 and cc3—the time between 2-cell and 3-cell stage, and between 3-cell stage and 5-cell stage, i.e., duration of the second and third cell cycle, respectively; s2 and s3—the cleavage timing between progeny in the different cell generations, i.e,. synchronicity of second and third rounds of cleavage divisions, respectively; sM—compaction and cavitation, the period during which the embryo remains in the morula stage. On all graphs, the total number of embryos analyzed per genotype is indicated in parentheses. On the boxplots, the middle lines represent medians, the cross shows the mean values, the hinges indicate the interquartile ranges, the whiskers represent the minimum and maximum values within the group and the dots show outliers. Statistical analysis was performed using the Kruskal–Wallis test with post hoc Dunn test, separately for each morphokinetic parameter. ^*^P < 0.05; ^**^P < 0.01; ^***^P < 0.001. (C) Confocal images of blastocysts immunostained with antibodies against CDX2 (TE), SOX17 (PE), SOX2 (EPI), with chromatin stained with Hoechst. Scale bars: 20 μm. (D–E) Percentage contribution of primary cell lineages, negative and bipotential cells within (D) blastocyst and (E) inner cell mass in blastocysts (*P* > 0.05, Kruskal–Wallis test). Abbreviations: trophectoderm (TE), inner cell mass (ICM), primitive endoderm (PE), epiblast (EPI), negative (neg.; CDX2- SOX17- SOX2-) cells, bipotential (bipot.; SOX2+ SOX17+) cells. See also Movie 1. (F) Mitochondrial membrane potential assessed by JC-1 staining. The JC-1 monomers indicate low mitochondrial membrane potential, while JC-1 aggregates form in mitochondria with high membrane potential. The fluorescent photographs are maximum orthogonal projections from ten z-stacks, whereas the bright field presents a single subset from the middle of the oocytes. Scale bars: 50 μm. (G) Ratios of JC-1 red to green fluorescence intensities, indicating mitochondrial activity. Statistical analysis using Mann–Whitney U-test revealed a significant difference in the activity of mitochondria (^***^P < 0.001).

To investigate the maternal effect of *Inhba* knockout during the early stages of preimplantation development, we crossed *Inhba*^KO/LoxP^  *Zp3*-Cre females with *Inhba*^KO/LoxP^ males, generating embryos with maternal activin A knockout (*n* = 35) and embryos with both maternal and zygotic protein knockout (*n* = 26). Control *Inhba*^LoxP/LoxP^ embryos (*n* = 101) were obtained by crossing *Inhba*^LoxP/LoxP^ females with males of the same genotype. To exclude the influence of exogenous activin A from the female reproductive tract, we isolated embryos at the zygote stage and monitored their development to the blastocyst stage using time-lapse imaging. The analyzed morphokinetic parameters are presented in Figure 3A and B. Our measurements revealed significant delays in *Inhba* m-KO embryos compared to control embryos in key developmental milestones, including the timing of the nuclear envelope breakdown of pronuclei (tNEBD), cleavage divisions (t2–t8), compaction (tM), and cavitation (tC) (Figure 3A; Movie 1). Furthermore, m-KO embryos exhibited an extended duration of the third cell cycle, specifically from the 3-cell to the 5-cell stage (cc3; *P* < 0.001, Kruskal–Wallis test with post hoc Dunn test; Figure 3B), as well as reduced synchrony of the third cleavage round (s3; *P* < 0.01, Kruskal–Wallis test with post hoc Dunn test; Figure 3B). In contrast, parameters such as hatching time (tH), the duration of the first mitotic division (m1), the second cell cycle (cc2), second synchrony (s2), and the time spent in the morula stage (m1), were similar between these two groups (*P* > 0.05, Kruskal–Wallis test; Figure 3A and 3B).

To investigate whether embryo-produced activin A is required in the absence of maternally deposited activin A, we analyzed the development of maternal/zygotic knockout *Inhba* mz*-*KO embryos (*n* = 26). Our results revealed that simultaneous depletion of both maternal and zygotic activin A does not impose additional harm on embryo development, as most of the analyzed morphokinetic parameters were comparable between *Inhba* m-KO and *Inhba* mz-KO embryos (Figure 3A and B). The only notable difference between these two groups was an extended second cell cycle in *Inhba* mz-KO embryos compared to *Inhba* m-KO embryos (cc2; *P* < 0.05, Kruskal–Wallis test with post hoc Dunn test; Figure 3B). It is noteworthy that both m-KO and mz-KO embryos progress through most morphokinetic events at a slower rate compared to the control group (Figure 3A and B).

It should be noted that, although the observed differences are statistically significant, their effect size is small. This raised the question of whether the delayed development of m-KO embryos holds biological relevance. To address this, we analyzed the efficiency of control and m-KO embryos to develop to the blastocyst stage. We found no difference in the developmental success rate between *Inhba* m-KO, *Inhba* mz-KO, and control embryos. Among 35 *Inhba* m-KO and 26 *Inhba* mz-KO zygotes subjected to time-lapse imaging, 30 and 23, respectively, reached the blastocyst stage (85.7% and 88.5%). This proportion was similar to that of control embryos (90/101; 89.1%; *P* > 0.05, Fisher Exact test).

Furthermore, we detected the presence of epiblast (EPI; SOX2), primitive endoderm (PE; SOX17), and trophectoderm (TE; CDX2) markers, to evaluate the impact of retarded cleavages on cell lineage specification (Figure 3C). Surprisingly, neither *Inhba* m-KO nor *Inhba* mz-KO embryos showed significant differences in the number and percentage of cells contributing to the EPI, PE, and TE lineages when compared to control blastocysts, or each other (*P* > 0.05; Kruskal–Wallis test; Figure 3D and E).

Thus, our results demonstrate that the knockout of maternal activin A affects the dynamics of mouse preimplantation development without compromising the embryo’s ability to reach the blastocyst stage or form the correct first cell lineages.

### Maternal activin A deficiency affects mitochondrial activity in MII oocytes

Given that early embryonic development is an energy-consuming process, we hypothesized that the reduced cleavage rates observed in *Inhba* m-KO embryos may result from impaired mitochondrial function. Mitochondria are maternally inherited organelles that generate adenosine triphosphate (ATP) through high efficiency oxidative phosphorylation, providing the energy essential for driving cleavage divisions, cell cycle progression, and cytoskeletal remodeling (reviewed in [19]).

To evaluate mitochondrial function as a key regulator of metabolic activity, we employed JC-1 (5,5′,6,6′-tetrachloro-1,1′,3,3′-tetraethyl-imidacarbocyanine iodide) [20], a fluorescent, membrane-permeable dye that selectively accumulates in mitochondria. JC-1 exhibits a potential-dependent fluorescence shift: it emits green fluorescence in its monomeric form under low membrane potential, and red fluorescence in its aggregated form under high membrane potential. Since mitochondrial polarization reflects the capacity for oxidative phosphorylation and ATP generation, the red/green fluorescence ratio serves as a proxy for mitochondrial health and bioenergetic activity.

Using this assay we found that mitochondrial membrane potential was significantly reduced in *Inhba* m-KO MII oocytes compared to controls (0.498 vs 0.947; *P* < 0.001; Mann–Whitney U-test; Figure 3F and G), indicating that the absence of maternal activin A leads to mitochondrial dysfunction.

### Mouse preimplantation embryos lack zygotically produced activin A

As maternal knockout embryos and maternal/zygotic knockout embryos do not differ significantly in the course and rate of development, and zygotic knockout alone does not exhibit any preimplantation knockout phenotype [14], we next sought to verify previous reports on the expression of mRNA for activin A before implantation. As the level of *Inhba* gene expression in oocytes and preimplantation mouse embryos was below the detection range using RT-qPCR (data not shown), we considered ddPCR as the next best available approach. To exclude the possibility that the mRNA levels could vary due to environmental conditions or the expression of other activin isoforms, we determined the number of *Inhba* as well as *Inhbb* transcripts in embryos developed both in vivo and in vitro.

**Figure 4:**
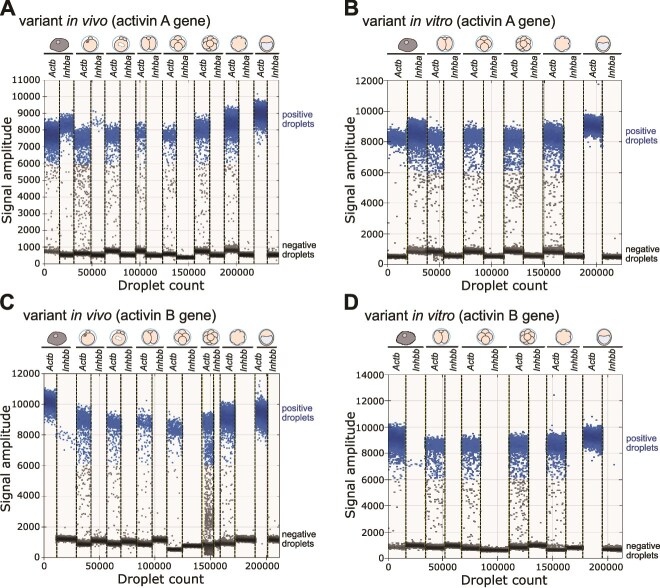
Mouse preimplantation embryos lack zygotically produced activin A. The results of ddPCR analysis of *Inhba* and *Inhbb* mRNA levels in wild-type MII oocytes, zygotes, 2-cell, 4-cell, 8-cell, morula-, and blastocyst-stage embryos (pooled samples of 26 to 70 embryos per stage; see Methods section) and a positive control—wild-type ESCs. The graphs illustrate the fluorescent amplitude observed for individual transcripts: the reference housekeeping gene *Actb* (β-actin gene) and *Inhba* (activin A gene) or *Inhbb* (activin B gene). Dots in the upper cluster represent positive droplets in which amplification of the target fragment occurred, while dots in the lower cluster correspond to the negative droplets without a PCR product of: (A) *Inhba* in ESCs, oocytes, zygotes, and embryos developed in vivo; (B) *Inhba* in ESCs and embryos developed in vitro; (C) *Inhbb* in ESCs, oocytes, zygotes, and embryos developed in vivo; (D) *Inhbb* in ESCs and embryos developed in vitro.

We confirmed the presence of an *Inhba* mRNA in mouse ESCs (Figure 4A and B). Surprisingly, we discovered that 2-, 4- and 8-cell, morula, and blastocyst stage embryos contained no detectable copies of *Inhba* transcripts, while only a low fluorescence signal of *Inhba* was present in MII oocytes and zygotes. These results were consistent regardless of whether the embryos developed in vivo or in vitro (Figure 4A and B, respectively). The low levels of *Inhbb* mRNAs were detected in ESCs, while these transcripts were absent both in MII oocytes and embryos at all stages, whether developed in vivo or in vitro (Figure 4C and D).

**Figure 5:**
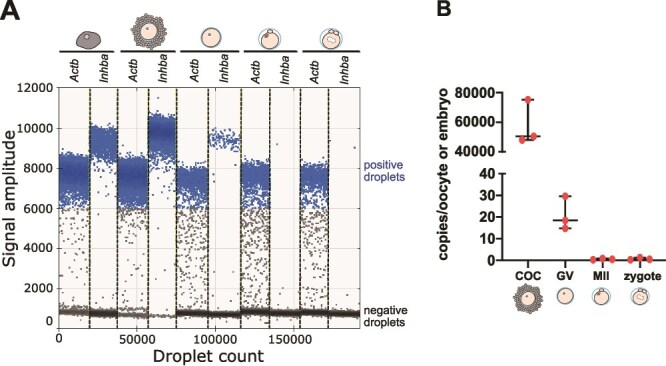
Maternal *Inhba* transcripts are present mostly in follicular cells and GV oocytes. (A) Validation of the presence of reference housekeeping gene *Actb* (β-actin gene) and *Inhba* (activin A gene) transcripts in ESCs, GV oocytes surrounded by cumulus follicular cells, denuded GV oocytes, MII oocytes, and zygotes. The graphs illustrate the fluorescent amplitude of target genes, with positive droplets (containing gene transcript) in the upper cluster and negative droplets (without target signal) in the lower cluster; (B) *Inhba* copy numbers per COC, denuded GV oocyte, MII oocyte or zygote analyzed by ddPCR. The middle lines represent median and the whiskers indicate the interquartile ranges. Each ddPCR reaction was performed using three biological replicates.

Given the low fluorescence signal of *Inhba* transcripts in MII oocytes, and the premise that the follicular cells prominently express *Inhba* subunit mRNAs [21], we next conducted an in-depth examination of the number of copies of *Inhba* transcripts in GV oocytes surrounded by a corona radiata (cumulus-oocyte complex; COC), as well as in GV oocytes, MII oocytes, and zygotes devoid of follicular cells. Surprisingly, we found that *Inhba* gene is expressed more abundantly in the follicular cells of the corona radiata than in the oocytes themselves, as COC has over 2700-fold more *Inhba* transcripts than denuded GV oocyte (50 560 vs 18.40 copies per oocyte, respectively; Figure 5A and B). Furthermore, the copy number of maternal *Inhba* decreases from the GV oocyte stage onward, with only a small number of *Inhba* transcripts detected in MII oocytes and zygotes (0.48 copies per oocyte/zygote for both; Figure 5A and B).

Thus, our results reveal the lack of *Inhba* transcripts of zygotic origin in mouse preimplantation embryos, with maternal transcripts present only in the follicular cells, oocytes and zygotes. Furthermore, we provide evidence that *Inhbb* mRNA is absent in oocytes and embryos before implantation.

## Discussion

To date, several dozen maternal-effect factors have been identified in mice, providing valuable insights into the molecular mechanisms underlying early embryonic development [17]. Given that an early embryo relies on mRNAs and proteins deposited in the oocyte during oogenesis until activation of the zygotic genome, we sought to determine whether maternal activin A expression might mask the phenotype of the zygotic knockout. To this end, we deleted the *Inhba* gene from the oocytes before completing the first meiotic division using the *Zp3*-Cre/LoxP strategy. We found that a lack of maternal activin A does not prevent normal preimplantation development to the blastocyst stage. However, it affects the rate of divisions, leading to a delay in developmental progression. The observed alterations in the cleavage kinetics may be associated with the function of activin A in cell cycle regulation [22]. Studies suggest that activin plays a dual role as a molecule capable of both promoting and inhibiting cell proliferation. It has been reported to stimulate the proliferation of many different target cells of various origins, such as human lung fibroblasts, mouse keratinocytes, and rat spermatogonial cells [23–25]. Additionally, during oogenesis, activin A promotes the proliferation of oogonia [26] and follicular cells [27, 28]. On the other hand, activin A has demonstrated the ability to inhibit cell proliferation in several cell types, including human breast cancer cell lines [29], prostate cancer cell lines [30], vascular endothelial [31], vascular smooth muscle cells [32], and 3T3 mouse fibroblasts [33]. These diverse actions of activin A indicate the existence of regulatory mechanisms that govern its bioavailability, ensuring appropriate and timely expression in various tissues.

To explore the cause of the reduced rate of embryonic cleavage in m-KO and mz-KO embryos, we considered the possibility of insufficient energy production due to mitochondrial dysfunction. To investigate this, we assessed the metabolic profile of oocytes using JC-1 staining, which enabled evaluation of mitochondrial membrane potential (ΔΨm).

We found that *Inhba* m-KO oocytes exhibited a significant decrease in mitochondrial membrane potential, suggesting that the absence of maternal activin A negatively affects mitochondrial function. Given that ΔΨm is a key indicator of active oxidative phosphorylation, this reduction suggests a diminished capacity to produce ATP, a critical energy currency required for numerous post-fertilization processes, including microtubule polymerization, chromosome segregation, membrane biosynthesis, and cell cycle progression [19].

While no literature data noted the direct connection between activin A and mitochondrial activity in oocytes, Li et al. (2009) reported the influence of this protein on mitochondrial metabolism in adipose tissues [34]. Mice with a reduced expression of *Inhba* exhibit increased mitochondrial function caused by excessive uncoupling of the mitochondrial inner membrane, leading to enhanced mitochondrial biogenesis and elevated oxygen consumption as a failed remedy. While these findings are not oocyte-specific, they nonetheless suggest that activin A can indirectly modulate mitochondrial function.

In mammalian embryos mitochondria are almost exclusively maternally inherited [35]. Notably, since mitochondrial replication does not occur until after the hatched blastocyst stage, early cleavage-stage embryos rely entirely on the function of the mitochondria inherited from the MII oocyte. Consequently, any functional deficit at the oocyte stage can compromise cell bioenergetics and disrupt key developmental processes during early embryogenesis. An adequate reserve of mitochondrial ATP is particularly critical during the oocyte-to-zygote transition and the subsequent rapid cleavage divisions.

Our findings are consistent with previous observations in human oocytes demonstrating that impaired mitochondrial activity is associated with abnormal embryo development. Specifically, low mitochondrial membrane potential has been linked to delayed embryonic cleavage [36] and chaotic chromosomal mosaicism [37]. Moreover, energy substrate depletion in culture media was shown to reduce mitochondrial membrane potential and slow, but does not completely arrest, development of human embryos [38]. Complementary data from mouse models reinforce this link between mitochondrial dysfunction and impaired cleavage dynamics during preimplantation development [39, 40]. Moreover, Wang et al. (2009) reported that lower ΔΨm and ATP content strongly correlate with a 2-cell developmental block in mouse embryos, underscoring the critical importance of mitochondrial activity for successful progression of development [41].

Taken together, these data support our findings that the maternal activin A deficiency leads to a reduction in mitochondrial activity within the oocyte, resulting in energy deficits that compromise timing of cleavage divisions. Nevertheless, despite the early developmental delay, *Inhba* m-KO and mz-KO embryos are capable of reaching the blastocyst stage with the correct number and distribution of cell lineages. This indicates that activin A plays a regulatory, rather than a pivotal, role during the preimplantation period of development, perhaps fine-tuning the metabolic state of the oocyte to optimize the timing and coordination of early embryonic events.

Given that activin A has been detected in oocytes [1, 4], and that mitochondrial activity is essential for successful oocyte maturation (reviewed in [42]), we were curious whether this protein plays a role in regulating meiotic progression. To this end, we tested the requirement of maternal activin A for IVM and, unexpectedly, revealed that conditional deletion of *Inhba* in growing oocytes resulted in enhancement of meiotic resumption and maturation. The involvement of activin A in the regulation of oocyte meiotic maturation has been studied across mammalian species, yielding conflicting results. It was shown that while activin A stimulates IVM in human and rhesus monkey oocytes [43, 44], it does not affect bovine oocytes [45] and exhibits an ambiguous effect on rat oocytes [46, 47]. These experiments relied on treating GV oocytes with exogenous activin A to mimic the paracrine action of activin A produced by follicular cells, which are known to express high levels of this protein and affect oocyte maturation [21] through gap junction communication. In contrast, our research, which focused on demonstrating the autocrine effect of activin A, suggests that maternal activin A accumulated in the oocytes during oogenesis is not essential for their maturation. Unexpectedly, oocytes with maternal *Inhba* knockout exhibited higher IVM efficiency than control oocytes. However, this difference likely stemmed from reduced IVM efficiency in the control group. As our experiments involved unstimulated with exogenous gonadotropins females, variations in their phases of the oestrous cycle or the transcriptional status of the oocytes (non-surrounded and surrounded nucleolus) [48, 49] may have influenced the outcomes. Despite the apparent dispensability of activin A for IVM, we cannot rule out its potential significance before the primary follicle stage, when *Cre* transgene expression is initiated through activation of the *Zp3* promoter.

The experiments described above, along with our previous study [14], and the recent report demonstrating the dispensability of maternal and zygotic Smad4 [50], a crucial effector of canonical activin signaling, raised doubts about the functionality of maternal and zygotic activin A in mouse oocytes and preimplantation embryos. To resolve discrepancies in the literature regarding the expression of activin A and B during these developmental stages, we decided to revisit this question using ddPCR, which is a method that enables highly sensitive and precise detection of low-abundance transcripts. Our results indicate that *Inhbb* mRNA is exclusively present in ESCs, while *Inhba* transcripts are detectable in ESCs, follicular cells, oocytes, and zygotes. Neither *Inhba* nor *Inhbb* genes are expressed at any subsequent stages of mouse preimplantation development, regardless of whether the embryos developed in vivo or were cultured in vitro. This finding strikingly contrasts with the results of previous studies demonstrating the presence of *Inhba* mRNA or activin A zygotic protein in mouse and human preimplantation embryos using the RT-PCR and immunofluorescence methods [1–4]. In traditional RT-PCR, detecting low-abundance targets like *Inhba* is often unreliable or yields erroneous results, as measurements approach the technique’s sensitivity. In addition, ddPCR surpasses the traditional real-time PCR method by minimizing reaction variability, making it more reliable. Concurrently, our ddPCR results partially confirm the conclusions drawn from previous transcriptomic analysis [6, 8], reinforcing the validity of these reports.

The absence of *Inhba* transcripts in preimplantation embryos suggests that it is not expressed during the maternal-to-zygotic transition at the 2-cell embryo. Thus, we infer that the mRNA detected in zygotes is of maternal origin. Unfortunately, due to the unavailability of a reliable antibody, we were unable to verify the presence of maternal protein in the subsequent preimplantation stages. Previous reports demonstrated that most maternal gene products are destroyed at the 2- or 8-cell stages [51]. However, some of them have been observed to persist up to the blastocyst stage, including E-cadherin, β-catenin, the nucleopore protein CAN/nup24, Sox2, Mater, and Max [52–57]. We cannot rule out that activin A protein, produced from maternal transcripts, persists until the blastocyst stage and regulates cleavage divisions, as demonstrated in our study. Our data indicate that tight regulation of local maternal activin A levels within the embryo is not a prerequisite for normal development to the blastocyst stage, but supports its proper progression.

## Materials and methods

### Mouse lines

To deplete maternal activin A from the oocytes, we used heterozygous *Inhba*^LoxP/KO^  *Zp3*-Cre females from B6;CBA-Inhba^em1Iimcb^/Inhba^em3Iimcb^ Tg(Zp3-cre)93Knw/Tar mouse line (MGI:8215005), derived by crossing mice with floxed *Inhba* gene and an *Inhba* functional knockout (KO), and carrying the Cre recombinase gene under the control of *Zp3* promoter. The *Inhba-LoxP* (B6;CBA-Inhba^em3Iimcb^/Tar; MGI:8215003) and *Inhba-*KO mouse lines (B6;CBA-Inhba^em1IIMCB^/Tar; MGI:8211126); previously described in [14], were generated using the CRISPR/Cas9 method in the Genome Engineering Unit of the International Institute of Molecular and Cell Biology (Warsaw, Poland). The Cre mouse line (C57BL/6-Tg(Zp3-cre)93Knw/J; MGI:2164742) was a gift from David Drutovic.

To obtain embryos, *Inhba*^LoxP/KO^  *Zp3*-Cre females were mated with *Inhba*^LoxP/KO^ males (from B6;CBA-Inhba^em1Iimcb^/Inhba^em3Iimcb^/Tar mouse line; MGI:8215004) (Figure 1). Excision of the floxed *Inhba* allele in the female germline by Cre recombinase leads to the complete depletion of activin A in all oocytes (*Inhba*^KO^ or *Inhba*Δ). Half of the resulting embryos are maternal/zygotic knockouts (*Inhba*^KO/KO^ or *Inhba*^Δ/KO^, henceforth referred to as mz*-*KO) and half of the embryos that have floxed but functional paternal *Inhba* allele are maternal knockouts (*Inhba*^KO/LoxP^ or *Inhba*^Δ/LoxP^, henceforth referred to as m*-*KO). As a control, we used *Inhba*^LoxP/LoxP^ homozygotes. All transgenic mouse lines were maintained in a C57BL/6/Tar x CBA/Tar mixed genetic background. For ddPCR analysis, we used wild-type F1(C57BL/6/Tar x CBA/Tar) females mated with F1(C57BL/6/Tar x CBA/Tar) males.

All mouse lines were bred in the Animal Facility of the Faculty of Biology, University of Warsaw. Animal experiments were approved by the Local Ethics Committee for Experimentation on Animals no. 1, Warsaw, Poland (permissions no 1336/2022, 1523/2023, 1577/2024) and were conducted under the ARRIVE guidelines and national regulations.

### Mouse genotyping

Mouse DNA was isolated using the HotShot method [58]. Tissue samples for isolation included a fragment of tail taken post-mortem from fetuses or discarded ear punches from adult individuals. The collected tissues were incubated for 25 min in 120 μl of alkaline lysis solution (pH = 12), preheated to 95°C, followed by cooling on ice for 5 min. After centrifugation of the samples at 400 g for 10 s, lysis was inhibited by adding 120 μl of neutralizing solution (pH = 5). The isolated DNA was either used immediately or stored at 4°C. For PCR, 1 μl of DNA extract was added to 9.5 μl of PCR mix containing Phusion HS II polymerase, Green HF buffer (ThermoFisher Scientific), 200 μM dNTPs, nuclease-free water, and 0.5 μM primers. To detect *Inhba* KO allele, forward 5′- CACAAACCTACAGCACTGAC-3′ and reverse 5’-CCACTTTACCCACATGAAGC-3′ primers were used, and amplification proceeded under the following conditions: an initial denaturation at 98°C for 3 min and 13 s, followed by 35 repeated cycles, with 98°C for 13 s, 63°C for 17 s and 72°C for 9 s, and final extension at 72°C for 5 min. Genotyping of *Inhba* LoxP allele proceeded with 5’-TGCAGAACTGAGGTCTCTGC-3′ and 5’-GACCACTGAGTTGAGAAGGG-3′ primers, according to the programme: 98°C for 3 min, followed by 35 repeated cycles, with 98°C for 13 s, 63°C for 17 s and 72°C for 7 s, and 72°C for 5 min. The PCR reaction for the *Zp3* Cre allele was performed using 5’-TATTCGGATCATCAGCTA-3′ and 5’-GGTGGGAGAATGTTAATC-3′ primers, under the following conditions: 95°C for 2 min, followed by 30 repeated cycles, with 95°C for 30 s, 56°C for 30 s and 68°C for 20 s, and final extension at 68°C for 2 min. Subsequently, amplicons of *Inhba* KO and *Inhba* LoxP alleles were digested at 37°C for 12 min with EcoRI and BamHI enzymes, respectively, followed by electrophoresis separation in a 1.5% agarose gel. After digestion, the *Inhba* KO allele gives two products of 332 and 171 bp size, whereas the wild-type allele results in a single 482 bp product. The *Inhba* LoxP allele is identified by the presence of two products: 191 bp and 195 bp, while the wild-type allele gives a single 346 bp band. The *Zp3* Cre is identified by the presence of a single 139 bp product.

### Isolation of germinal vesicle oocytes and in vitro maturation

To collect GV oocytes, six 2–4-month-old randomly cycling *Inhba*^KO/Flox^  *Zp3*-Cre and 6 *Inhba*^LoxP/LoxP^ females were sacrificed. Ovaries were isolated and COCs were released from ovarian antral follicles by puncturing with a needle into the M2 medium. Fully grown GV oocytes were cleared of follicular cells by gentle pipetting. In vitro maturation was performed for 15–16 h in a pre-balanced M16 medium under mineral oil (Fujifilm) at 37.5°C and 5% CO_2_ in the air. After 16 h of the culture of oocytes in vitro, the ability of the oocytes to reach metaphase II was rated based on the extrusion of a first polar body (PB1) and the GV breakdown (GVBD). The structure of the meiotic spindle was visualized by β-tubulin immunostaining and chromatin labeling. IVM was performed in six independent experiments with the use of six females of each genotype.

### Isolation of zygotes for time-lapse imaging

Mouse zygotes were isolated from the ampullae of oviducts of superovulated *Inhba*^KO/Flox^  *Zp3*-Cre and *Inhba*^LoxP/LoxP^ females (10 IU of PMSG followed by 10 IU of hCG; Intervet) mated with *Inhba*^KO/Flox^ or *Inhba*^LoxP/LoxP^ males in the same mixed genetic background (C57BL/6/Tar x CBA/Tar). Females, in which vaginal plugs were detected, were sacrificed 24 h after the hCG injection. Zygotes were recovered and cleared of follicular cells using hyaluronidase (300 μg/ml, Sigma-Aldrich). All embryos were collected from four *Inhba*^KO/Flox^  *Zp3*-Cre and four *Inhba*^LoxP/LoxP^ females in four independent experiments.

### Time-lapse imaging and morphokinetic analysis

The preimplantation development of *Inhba* m-KO and *Inhba* mz-KO zygotes was monitored using time-lapse recording (every 10 min) under the PrimoVision imaging system enclosed in a standard embryo culture incubator maintaining constant culture conditions (37.5°C, 5% CO_2_). The embryos were cultured in 16-well dishes (Vitrolife) with 30-μl droplets of M16 medium (Sigma Aldrich) under mineral oil for 4 days. The acquired images were analyzed using ImageJ software to assess morphokinetic parameters, which included: 1) t_NEBD_—the time between the hCG injection and the nuclear envelope breakdown of pronuclei; 2) t_2_ to t_8_—the periods between the hCG injection and the moment when the embryo reaches a specific number of cells; 3) t_comp_ and t_cavit_—the time between the hCG injection and initiation of compaction and cavitation, respectively; 4) m_1_—the duration of the 1st embryonic cycle, i.e., the period between the disappearance of pronuclei and 2-cell stage; 5) cc_2_ and cc_3_—the duration of the cell cycle for 2- or 4-cell stage blastomeres, respectively; 6) s_2_ and s_3_—the synchronicity of 2nd and 3rd rounds of cleavage divisions, respectively; 7) s_M_—a period between compaction and cavitation when embryo remains in morula stage. To ensure the accurate distribution of genotypes for the isolated embryos, zygotes that did not fit into the recording dish were cultured outside the imaging area, followed by processing along with the recorded embryos. Once filming was completed, all the embryos were individually fixed while preserving their identities. Subsequently, embryos that reached the blastocyst stage were stained for cell lineage markers and after analysis under a confocal microscope, all blastocysts were individually genotyped. Embryos that were arrested during development were fixed and genotyped without further analysis.

### Immunofluorescent staining, confocal microscopy, and image processing

Oocytes and blastocysts were individually fixed in 4% paraformaldehyde (ThermoFisher Scientific) in Ca^2+^ and Mg^2+^-free PBS (Biomed) for 30 min, permeabilized with 0.5% Triton X-100 (Sigma-Aldrich) for 20 min, and incubated in 3% BSA (Sigma-Aldrich) with 0.01% sodium azide (Honeywell Fluka) at 4°C. Oocytes were immunostained for β-tubulin using a mouse monoclonal antibody conjugated to FITC (1:50; 2 h at room temperature or overnight at 4°C; Sigma-Aldrich, F2043). For blastocysts, the following primary antibodies were used: mouse monoclonal antibody against CDX2 (1:50, BioGenex, MU392A-UC, RRID:AB_2923402), rabbit polyclonal antibody against SOX2 (1:100, Abcam, ab97959, RRID:AB_2341193), goat polyclonal antibody against SOX17 (1:100, R&D Systems, AF1924, RRID:AB_355060). After 24 h of incubation with primary antibodies at 4°C, the blastocysts were washed three times for 15 min each in Ca^2+^ and Mg^2+^-free PBS and then incubated for 1.5 h at room temperature in a mixture of matching secondary antibodies: donkey anti-mouse IgG conjugated with Alexa Fluor 594 (1:200, Invitrogen, A21203, RRID:AB_2535789), donkey anti-rabbit conjugated with Alexa Fluor 647 (1:200, Invitrogen, A31573, RRID:AB_2536183), donkey anti-goat IgG conjugated with Alexa Fluor 488 (1:200, Invitrogen, A11055, RRID:AB_2534102). All primary and secondary antibodies were diluted in 3% BSA. Following further washes in PBS, chromatin was stained with Hoechst 33342 (20 μg/ml in PBS, ThermoFisher Scientific) for 20 min at 37°C. The confocal analysis of oocytes and blastocysts was performed on glass-bottom dishes (MatTek Corporation). Images were acquired with a Nikon Ti2-U microscope equipped with a focus motor assembly (Prior Scientific Instruments), the rescan confocal microscopy module RCM1 (Confocal.nl), an ORCA-Flash4.0 LT + camera (Hamamatsu) and iChrome CLE 50 laser engine (Toptica) using 10X and 25X immersion objectives (Nikon). For each blastocyst, z-stacks were collected with 2.5 μm intervals between optical sections. Confocal images were analyzed using ZEN 2.3 (blue edition) software, and blastocyst cell counting was semiautomated with Imaris software. For oocytes, z-stacks were collected with 1.5 μm intervals between optical sections. To visualize all sides of the meiotic spindle, the background noise of the images was reduced using deconvolution tools of Huygens Essential software, and the resulting images were analyzed as 3D z-stack projections using Imaris software.

### Detection of mitochondrial activity

MII oocytes were isolated from the ampullae of oviducts of superovulated *Inhba*^LoxP/KO^  *Zp3*-Cre and *Inhba*^LoxP/LoxP^ females (10 IU of PMSG followed by 10 IU of hCG; Intervet). To assess mitochondrial activity, oocytes were incubated in a 2 μM solution of JC-1 dye (ThermoFisher Scientific) in M2 with BSA for 30 min at 37.5°C, rinsed, and transferred to a glass-bottom dish for imaging. JC-1 is an indicator of mitochondrial activity, as it accumulates in mitochondria in a membrane potential-dependent manner: at low mitochondrial membrane potential, JC-1 remains in its monomeric form, emitting green fluorescence, whereas at high potential, it forms aggregates that emit red fluorescence. Imaging was performed using a Zeiss Axio Observer.Z1 microscope (Zeiss) equipped with an environmental chamber and an AxioCam HRm camera. A total of 10 optical sections were captured along the z-axis, with a spacing of 5.5 μm apart. Excitation filters of 450–490 nm and 538–562 nm were used for the green and red channels, respectively, and emission was collected at 500–550 nm (green) and 570–640 nm (red). The light source intensity was held at 5% and exposure times were set to 13 ms for the green channel and 45 ms for the red channel (4x4 binning). Image analysis was performed using the ImageJ software. Mitochondrial membrane potential was determined as the ratio of red to green fluorescence intensity, calculated from the sum of signal intensities across all z-stack layers. In each experiment, the mean fluorescence intensity of oocytes was normalized to that of control oocytes, which were stained and imaged simultaneously. Mitochondrial activity assessment was performed in three independent experiments with the use of four females of each genotype.

### Embryo genotyping

At the end of each experiment, DNA from individual embryos was extracted using the Extract-N-Amp Tissue PCR Kit (Sigma Aldrich) in a 5.5 μl mixture of Extraction and Tissue Preparation Solutions (4:1). Lysis was carried out at 56°C for 30 min, 24°C for 5 min, and 95°C for 5 min. Then 4.4 μl of N-Solution was added to each sample to stop the reaction. The PCR reaction was conducted using a Fast Cycling PCR Kit for the detection of *Inhba* KO allele and a Multiplex Plus PCR Kit (Qiagen) for the identification of functional and excised *LoxP* alleles. Crude DNA extracts were added to the PCR reaction mix containing the manufacturer’s PCR Mix, Q solution, nuclease-free water, and primers. The *Inhba* KO allele was amplified using 0,5 μM 5’-CTTTGGCTGAGAGGATTTCTG-3′ and 5’-CCACTTTACCCACATGAAGC-3′ primers. The PCR program was as follows: 95°C for 4 min, followed by 46 repeated cycles at 95°C for 30 s, 58°C for 45 s, 68°C for 45 s, and 72°C for 10 min. To identify the *Inhba* LoxP allele and the excised flanked *Inhba* gene fragment, 0,2 μM 5’-GCAGGCATCCAGGGTTTTC-3′, 5′- GACCACTGAGTTGAGAAGGG-3′ and 5’-CCACTGTCTTCTCTGGACTCTC-3′ primers were used, and PCR reaction proceeded according to program: 95°C for 5 min, followed by 40 repeated cycles, with 95°C for 30 s, 57°C for 90 s and 72°C for 30 s, and final extension at 68°C for 10 min. Then amplicons of *Inhba* KO and *Inhba* LoxP alleles were digested at 37°C for 12–20 min with EcoRI and BamHI enzymes, respectively, followed by electrophoresis. Genotyping of the *Inhba* KO allele after digestion resulted in two products of 128 and 171 bp size, while the wild-type allele produced a single 277 bp product. The functional *Inhba* LoxP allele was identified by the presence of two products, 236 bp and 195 bp after digestion, while the excised by recombinase Cre flanked exon 2 of *Inhba* gene (*Inhba*Δ) generated a 274 bp product. At the same time, the *Inhba* KO allele (on exon 1) was visible as 391 bp band.

### MEFs derivation and culture

To obtain *Inhba* m-KO and mz-KO MEFs, E13 fetuses were harvested from *Inhba*^KO/LoxP^  *Cre*-Zp3 females after natural mating with *Inhba*^KO/LoxP^ males. Control MEFs were isolated by crossing wild-type mice. Briefly, fetuses were released from the fetal membranes and euthanized by decapitation. A tail fragment from each fetus was collected for genotyping. Subsequently, the corpora of fetuses were minced and digested in 0.25% trypsin-EDTA at 37°C for 30 min. The enzymatic process was stopped by adding MEF medium containing high glucose Dulbecco modified eagle medium, 10% fetal bovine serum, and 1% penicillin–streptomycin. After the mechanical dissociation of tissue into a single-cell suspension, the resulting cells were cultured in T-75 flasks until they reached confluence, and then they were frozen for storage.

### Measurement of activin A by ELISA

*Inhba* m-KO (*n* = 5), mz-KO (*n* = 2) and wild-type MEFs (*n* = 2) were seeded at a density of 4 x 10^5^ cells per 60-mm dish and cultured until near confluence. Following 48 h of incubation, the cells were washed once, and the medium was replaced with 4 ml of fresh serum-free medium for the next 24 h. The supernatant of each culture medium was then collected and centrifuged at 1500 rpm (10 min, 4°C) to remove debris. The concentration of mouse activin A in the culture medium of MEF cell lines was measured using the activin A Immunoassay (human/mouse/rat activin A Quantikine ELISA kit, R&D Systems, #DAC00B) following the manufacturer’s instructions.

### Collection of samples for droplet digital polymerase chain reaction

F1(C57BL/6/Tar x CBA/Tar) female mice (2–3 months old) were induced to superovulation as described above, and to obtain embryos, were mated with male mice of the same mixed genotype. The GV oocytes were isolated from the ovaries of eight females, 15–17 h after PMSG injection. Some oocytes were left with follicular cells (as COC; three biological replicates, each with a pool of 50 COCs per sample), while others were deprived of them by pipetting (three biological replicates, each with a pool of 50 oocytes per sample). Ovulated metaphase II oocytes were isolated 15–17 h post-hCG injection (phCG) and pooled from four females (50 oocytes per sample, three biological replicates). For the “in vivo” variant, the zygotes (24 h phCG; 50–55 zygotes per sample, four biological replicates from four females), 2-cell embryos (50 h phCG; 60 embryos per sample from two females), 4-cell embryos (54 h phCG; 70 embryos per sample from three females), 8-cell embryos (69 h phCG; 43 embryos per sample from two females), and morula-stage embryos (78 h phCG; 57 embryos per sample from three females) were collected from the oviducts of the females. Blastocyst-stage embryos were collected from the uterus of the females 96 h phCG (50 blastocysts per sample from three females). The hyaluronidase solution was used to remove the follicular cells from MII oocytes and zygotes. For the “in vitro” variant, zygotes were collected from the females treated as described above and cultured in KSOM medium for 51 h (until the 2-cell stage; 50 embryos per sample from two females), 54 h (4-cell stage; 50 embryos per sample from three females), 74 h (8-cell stage; 50 embryos per sample from three females), 80 h (morula; 26 embryos per sample from two females), and 117 h (blastocyst; 50 embryos per sample from three females). The 10 000 wild-type mouse ESCs per sample were used as a positive control.

### Isolation of RNA and droplet digital polymerase chain reaction assay

Total RNA was extracted and purified using the RNAqueous-Micro Kit (Thermo Fisher Scientific) according to the manufacturer’s instruction, with modifications of the reagent volumes. For each sample, a pool of oocytes, embryos, or ESCs was suspended in 50 μl of lysis buffer. The 62.5 μl of 100% ethanol was used for cell lysis, and a total volume of 20 μl solution was used for RNA elution from the filter. Reverse transcription was preceded by Oligo(dT)12–18 (ThermoFisher Scientific) primers hybridization to RNA at 70°C for 10 min in a T100 thermocycler (Bio-Rad). The synthesis of cDNA was performed using Superscript III reverse transcriptase (Invitrogen), RNA-ase inhibitor, dNTPs, RT buffer and DTT. Reactions were carried out in a thermocycler according to the program: 50°C for 50 min, followed by 70°C for 15 min. The resulting cDNA was diluted twofold with nuclease-free water.

Droplet digital polymerase chain reaction was performed in 20 μl reactions containing 10 μl ddPCR Supermix for Probes (Bio-Rad), 1 μl of specific TaqMan probes for β-actin (Mm01205647_g1), activin A (Mm00434339_m1) or activin B (Mm03023992_m1) (Applied Biosystem), 3 μl of PCR-grade water and 6 μl of cDNA. Droplets were generated using the QX200 Droplet Generator following the manufacturer’s Bio-Rad protocol. In this system, samples were partitioned into ∼20 000 nanoliter-sized droplets. PCR amplification was then performed with Bio-Rad C1000 Touch Thermo Cycler: Stage 1 (95°C, 10 min); Stage 2 (94°C, 30 s; 60°C, 1 min; repeat for 40 cycles); Stage 3 (98°C, 10 min). Droplets were analyzed for positive (containing target DNA) and negative (no target DNA) signals using the QX200 Droplet Reader, and results were analyzed with QuantaSoft software (version 1.7.40917; BioRad) providing absolute quantification of target DNA molecules as target copies/μl of reaction. Each ddPCR reaction was performed using three biological replicates of cDNA with each replicate generated from a pool of 50 oocytes or embryos, or 10 000 ESCs. The number of *Inhba* transcripts in a single ESC, oocyte or embryo was calculated as follows [59]:


$$ \frac{\mathrm{copy}\ \mathrm{number}/\mathrm{\mu} \mathrm{l}\times \mathrm{dilution}\ \mathrm{factor}\times 200\ \mathrm{\mu} \mathrm{l}}{\mathrm{number}\ \mathrm{of}\ \mathrm{ESCs}\ \mathrm{or}\ \mathrm{oocytes}\ \mathrm{or}\ \mathrm{embryos}\ } $$

### Statistical analysis

Statistical analysis was conducted using GraphPad Prism 10 software. All quantitative data were verified for normal distribution using the Shapiro–Wilk test, while the homogeneity of variances was assessed with Levene test. When the assumptions for the parametric test were not met, or the group size (n) was less than 30, comparisons between two groups were made using the Mann–Whitney U-test, and for multi-group comparisons, the Kruskal–Wallis test was performed. If applicable, Dunn test was used as a post hoc test. For qualitative data, e.g., efficiency of blastocyst formation between different genotypes, and IVM analysis, the Fisher Exact test was used. In all tests, the value of *P* = 0.05 was considered a significant threshold.

## Supplementary Material

Movie_1_ioaf189

## Data Availability

The data underlying this article are available in Dane Badawcze UW Repository, at 10.58132/MZV68L. This paper does not report original code. Any additional information required to reanalyze the data reported in this paper is available from the lead contact upon request.

## Data and resource availability

### Lead contact

Further information and requests for resources and reagents should be directed to and will be fulfilled by the lead contact, Aneta Suwińska (a.suwinska@uw.edu.pl).

## Materials availability

This study did not generate new unique reagents.
